# Validation of a stroke symptom questionnaire for epidemiological surveys

**DOI:** 10.1590/S1516-31802010000400010

**Published:** 2010-07-01

**Authors:** Ivana Makita Abe, Alessandra Carvalho Goulart, Waldyr Rodrigues Santos, Paulo Andrade Lotufo, Isabela Martins Benseñor

**Affiliations:** I PhD. Attending physician, Hospital Universitário (HU), Faculdade de Medicina da Universidade de São Paulo (FMUSP), São Paulo, Brazil.; II PhD. Epidemiologist, Hospital Universitário (HU), Hospital das Clínicas (HC), Faculdade de Medicina da Universidade de São Paulo (FMUSP), São Paulo, Brazil.; III MD. Neurologist, Hospital das Clínicas (HC), Faculdade de Medicina da Universidade de São Paulo (FMUSP), São Paulo, Brazil.; IV PhD. Professor of Clinical Medicine and Epidemiology, Faculdade de Medicina da Universidade de São Paulo (FMUSP), and Superintendent of Hospital Universitário (HU), Faculdade de Medicina da Universidade de São Paulo (FMUSP), São Paulo, Brazil.; V PhD. Attending physician, Hospital das Clínicas (HC), Faculdade de Medicina da Universidade de São Paulo (FMUSP), and Hospital Universitário (HU), Faculdade de Medicina da Universidade de São Paulo (FMUSP), São Paulo, Brazil.

**Keywords:** Stroke, Validation studies, Population surveillance, Prevalence, Cross-sectional studies, Acidente vascular cerebral, Estudos de validação, Vigilância da população, Prevalência, Estudos transversais

## Abstract

**CONTEXT AND OBJECTIVE:**

Stroke is a relevant issue within public health and requires epidemiological surveillance tools. The aim here was to validate a questionnaire for evaluating individuals with stroke symptoms in the Stroke Morbidity and Mortality Study (Estudo de Mortalidade e Morbidade do Acidente Vascular Cerebral, EMMA), São Paulo, Brazil.

**DESIGN AND SETTING:**

This was a cross-sectional study performed among a sample of the inhabitants of Butantã, an area in the western zone of the city of São Paulo.

**METHODS:**

For all households in the coverage area of a primary healthcare unit, household members over the age of 35 years answered a stroke symptom questionnaire addressing limb weakness, facial weakness, speech problems, sensory disorders and impaired vision. Thirty-six participants were randomly selected for a complete neurological examination (gold standard).

**RESULTS:**

Considering all the questions in the questionnaire, the sensitivity was 72.2%, specificity was 94.4%, positive predictive value was 92.9% and negative predictive value was 77.3%. The positive likelihood ratio was 12.9, the negative likelihood ratio was 0.29 and the kappa coefficient was 0.67. Limb weakness was the most sensitive symptom, and speech problems were the most specific.

**CONCLUSIONS:**

The stroke symptom questionnaire is a useful tool and can be applied by trained interviewers with the aim of identifying community-dwelling stroke patients, through the structure of the Family Health Program.

## RESUMO

**CONTEXTO E OBJETIVO:**

A doença cerebrovascular como parte da agenda de saúde pública necessita de instrumentos de vigilância epidemiológica. O objetivo foi validar um questionário para avaliação individual de sintomas de acidente vascular cerebral através do Estudo de Morbidade e Mortalidade no Acidente Vascular Cerebral (EMMA), São Paulo, Brasil.

**TIPO DE ESTUDO E LOCAL:**

Estudo transversal realizado em uma amostra de habitantes do Butantã, uma área na zona oeste da cidade de São Paulo.

**MÉTODOS:**

Em todos os domicílios de área adstrita de uma unidade básica de saúde, moradores com mais de 35 anos responderam questionário de sintomas de acidente vascular cerebral enfocando fraqueza de membros e facial, dificuldade em falar, alteração de sensibilidade e déficit de visão. Foram selecionadas 36 participantes aleatoriamente para exame completo com neurologista (padrão-ouro).

**RESULTADOS:**

Considerando todas as questões do questionário, a sensibilidade foi de 72,2%, a especificidade, 94,4% e os valores preditivos foram 92,9% (positivo) e 77,3% (negativo). As razões de verossimilhança foram 12,9 (positiva) e 0,29 (negativa) e o coeficiente *kappa* obtido foi 0,67. Fraqueza de membros foi o sintoma mais sensível e dificuldade para falar, o mais específico.

**CONCLUSÃO:**

O questionário de sintomas cerebrovasculares é um instrumento útil, e pode ser aplicado por entrevistadores treinados com intuito de identificar pacientes que sofreram evento cerebrovascular na comunidade através da estrutura do Programa Saúde da Família.

## INTRODUCTION

During the last two decades, several epidemiological papers have indicated that cerebrovascular diseases are a public health issue in Brazil.^1-16^ The impact of cerebrovascular diseases matters not only because of the burden of death^1-8^ and the costs of hospitalizations,^9^ but also because of the late effects from the disease, such as the degree of motor disabilities, post-stroke depression, reduced cognitive function and, consequently, the reduced quality of life among stroke survivors and their caregivers.^10-12^ However, mortality data has a natural limitation; classical prevalence surveys^13^ are relatively expensive; and incidence-based population studies^14-16^ are only suitable for small and middle-size towns. Recently, in parallel with cross-sectional studies using the traditional multistage cluster approach, new technologies for gauging the frequencies of diseases and disabilities have emerged, such as telephone surveys^17^ and household inquiries conducted by community health workers within primary care settings.^18^ One challenge in developing a cerebrovascular disease surveillance system will be to acquire appropriate tools, as shown in the World Health Organization stepwise approach to stroke surveillance (WHO Steps Stroke).^19,20^

Steps-Stroke is a strategy for improving the research data on stroke. It has the aim of investigating the impact of cerebrovascular diseases in relation to three steps in the disease process.^19,20^ Step 1 is a hospital-based phase in which determinants of the acute event are investigated, stroke subtypes are identified and the early and late case-fatality rates and disability rates are calculated by applying the modified Rankin questionnaire and the short version of Barthel’s index. Step 2 is a community-based fatal stroke event register for identifying cases in which the patient either did not seek medical care or died before hospitalization. Finally, step 3 is a community-based non-fatal stroke event register that was originally designed to investigate both the incidence and the prevalence of cerebrovascular diseases. Improvements in the methods for investigating the prevalence of stroke are crucial for quantifying the real burden of stroke. Such methods need to be suitable and feasible for carrying out sequential cross-sectional studies that address a particular healthcare issue, such as the number of stroke survivors and the level of disability due to the stroke. Thus, a questionnaire identifying stroke symptoms derived from the Steps Stroke approach, with scientific validation, that can be implemented repeatedly within primary care settings at low cost will be a useful instrument for planning local healthcare systems.

The Stroke Mortality and Morbidity Study (Estudo de Mortalidade e Morbidade do Acidente Vascular Cerebral, EMMA) was designed to apply the three phases of the WHO Steps Stroke approach in an area of the city of São Paulo.^21^ For the purposes of determining stroke prevalence, we translated, adapted, validated and applied a simple stroke symptom questionnaire that had been created by researchers working on the MONICA study (MONItoring Trends and Determinants of CArdiovascular Diseases) in order to examine cognitive function. This MONICA ancillary study was named Memory and Morbidity among Augsburg Elderly (MEMO).^22^ The present paper addresses the validation of this individual stroke symptom questionnaire for step 3, using an interview by a board-certified neurologist as the gold standard.

## METHODS

The WHO Steps Stroke approach, with its three steps, is the reference for the EMMA study. It was planned to investigate the burden of stroke among inhabitants of Butantã. This is an area in the western zone of the city of São Paulo, consisting of six districts and encompassing a population of approximately 420,000 inhabitants with one community hospital owned by the University and 14 affiliated primary care units (four of them running the Family Health Program). The community-based hospital implemented step 1 in May 2006. The analyses on mortality data for step 2 were completed in 2006. Step 3 was completed in 2008, within the coverage area of one primary care unit. Further data on the design and concept of the EMMA project can be obtained elsewhere.^21^

The primary care unit (Unidade Básica de Saúde, UBS) chosen for step 3 was in the Jardim São Jorge area. Approximately 15,000 people live in this area, and they receive healthcare under the Family Health Program, a federal health strategy in which a lay community health worker visits all the families in his/her area every month, under nurse/physician supervision. Six family physicians attend cases at this primary care unit, and each physician works with six community health workers. Each health worker has the task of visiting all the households in his/her area once a month. This area was previously charted when the program was implemented in the Butantã district, and it has been continually brought up to date when families move away or new families come to the area.

The questionnaire was based on the Memo Study,^22^ which was applied door-to-door or by mail to investigate the prevalence of stroke. The Portuguese version translated from English (PAL) is shown in Annex 1.

Data collection was done in two phases. Most of the families were contacted at the time of the monthly visit in February 2008. If no one in the family could be contacted in February 2008, a second visit in March or even a third visit in April-June 2008 was made. The community health workers were trained by a physician (IMA) to apply a questionnaire, in order to identify possible stroke patients at the monthly visits to families. The screening procedure was a face-to-face interview with one of the people living in the house, generally the mother/spouse, who answered questions about stroke symptoms on behalf of everyone over the age of 35 years who was living in the house. The form included five questions on stroke symptoms, in relation to limb weakness (arm or leg), facial weakness, speech problems, sensory disorders and impaired vision (Annex 1).

When someone in the house screened positive, the community health worker applied a longer questionnaire to the index subject on the same day or another day, asking again about stroke symptoms, sociodemographic characteristics, frequent comorbidities and use of medication. To facilitate comprehension, two pictures showing the appearance of facial weakness and the most common visual impairments in stroke patients were presented by the interviewer. The questionnaire also asked the patient whether he/she had had a stroke and, if the answer was yes, where he/she had been treated by a doctor, whether a computed tomography (CT) scan had been performed and what type of stroke the patient had had (Annex 2).

### Validation study

The entire protocol was approved by the Institutional Review Board of Hospital Universitário of the Universidade de São Paulo. The sample size for the validation study was calculated based on a estimated prevalence of a positive individual questionnaires of 25%, ranging from 5% to 45%, with a 95% confidence interval. The sample was divided into two groups: (1) with low or no risk of stroke and (2) with moderate to high risk of stroke. The sample size was calculated as 18 in each group. Thus, out of the 579 individuals who screened positive, 60 were randomly selected, including 25 into group 1 (low or no risk of stroke) and 35 into group 2 (25 with moderate risk and 10 with high risk of stroke). Of these 60 individuals,, 36 agreed to cooperate and signed the second research agreement.

We took the questionnaire result to be positive when the participant gave positive responses to two or more questions, either about stroke symptoms (out of five questions) or presence of stroke (one question), with confirmation from a physician; or when he/she gave at least three positive responses (out of the six questions), whether confirmed by a physician or not.

All the randomly selected participants were invited to come for a neurological interview in the hospital setting. They were contacted by phone or were visited by a member of the research team. All the neurological interviews were conducted by the same neurologist, who was blinded to the results from the questionnaire. Medical diagnoses of stroke were made based on the clinical interview, neurological examination and review of previous medical records, and on the individuals’ responses on the National Institute of Health Stroke Scale (NIHSS) and the Modified Rankin scale for evaluating the presence of disabilities.

### Diagnostic criteria

Stroke was defined in accordance with the WHO criteria as "an episode with sudden or rapid onset with focal brain dysfunction resulting from occlusive or hemorrhagic lesions of the vascular supply of the brain or global brain dysfunction which persisted for more than 24 hours and no apparent cause other than vascular origin".^19^ Stroke subtypes were defined based on information from the participants, medical records or analysis of previous CT scans.

### Statistical methods

The results were presented as proportions or means with standard deviation. The chi-square and analysis of variance (ANOVA) tests were used as appropriate. The sensitivity, specificity, positive and negative predictive values, accuracy and positive and negative likelihood ratios were calculated by comparing the responses to the questionnaire with the gold standard represented by the neurological interview and consultation of the previous hospital medical records. We calculated sensitivity, specificity, predictive values and likelihood ratios for the questionnaire overall and for each item singly. We used the Landis and Kock classification^23^ for the kappa coefficient to assess the level of agreement between the questionnaire and the neurological examination.

## RESULTS

Table 1 shows the general characteristics of the individuals with and without stroke in the sample. For two symptoms, impaired vision and facial weakness, there were no differences between individuals with stroke compared to those without stroke detected by the questionnaire. The scales were administered by the neurologist. There were statistically significant differences between the groups with regard to the presence of limb weakness, speech problems and sensory disorders, and in relation to the scores on the NIHSS and modified Rankin scales.

**Table 1.** Characteristics of participants with and without stroke according to questionnaire submitted to validation with a neurological interview and analysis of previous medical history

	Positive screening questionnaire		P
	Yes (n = 14)	No (n = 22)	
Age (years)	57.2 (11.1)	52.3 (12.3)	0.23
Women (%)	57.1	77.3	0.27
number of symptoms reported	2.2	0.4	< 0.0001
paralysis	78.6	9.1	< 0.0001
facial weakness	28.6	9.1	0.18
difficult to speech	35.7	0	0.005
sensory disorders	42.9	9.1	0.036
visual disturbances	35.7	13.6	0.22
medical history of stroke	78.6	0	< 0.0001
medical diagnosis	78.6	0	< 0.0001
medical plus computadorized tomography diagnosis	50.0	4.5	0.003
National Institutes of Health Stroke Scale	2.6	1.1	0.027
Modified Rankin Scale	0.86	0.14	0.001

Table 2 shows the sensitivity, specificity, positive and negative predictive value and positive and negative likelihood ratio results for all of the questionnaires, taking the established criteria into account. The results showed reasonable sensitivity and high specificity. The positive likelihood ratio was 12.9 and the negative likelihood ratio was 0.29. The questionnaire failed especially in mild cases of stroke in which the patients presented symptoms with low levels of disability or no disabilities at all. Four patients (18.2%) with no previous history of stroke were diagnosed as cases of stroke after neurological examination.

**Table 2.** Sensitivity, specificity, positive and negative predictive value, positive and negative likelihood ratio and kappa coefficient of the EMMA *(Estudo de Mortalidade e Morbidade do Acidente Vascular Cerebral)* questionnaire (*) for stroke diagnosis using clinical interview with a board certified neurologist as a gold standard.

		Stroke (neurological evaluation)		Total
		Yes	No	
EMMA Questionnaire (*)	Yes	13	1	14
	No	5	17	22
Total		18	18	36

Sensitivity = 72.2%, Specificity = 94.4%, Positive Predictive Value = 92.9%, Negative Predictive Value = 77.3%, Accuracy = 83.3%, Positive Likelihood Ratio = 12.9, Negative Likelihood Ratio = 0.29, kappa = 0.67.

(*) Portuguese version of the Memo Study (reference # 23). See annex 2.

Specific symptoms were considered in the analyses in Table 3, and all of them had higher specificity than sensitivity. In contrast to the positive likelihood ratio for the overall questionnaire, these ratios for each symptom alone were relatively lower. The assessment of interobserver variability, i.e. between the questionnaire criteria and the diagnosis made by the board-certified neurologist was calculated by means of the kappa coefficient. Using the Landis and Kock classification^25^ for the level of agreement shown by the kappa coefficient, we found that the level was "substantial" for the questions overall; "moderate" for "limb weakness" and for the category "at least one question addressing stroke symptoms"; "fair" for "facial weakness" and for "speech problems"; and "poor" for "sensory disorders" and "impaired vision".

**Table 3.** Performance of sensitivity, specificity, positive and negative predictive values, positive and negative likelihood ratios and kappa for each symptom separately and the use of just one question for stroke confirmation

Symptom	Limb weakness	Facial Weakness	Articulation problems	Sensorydisorders	Impaired vision	At least 1 question addressing stroke symptoms
Sensitivity ((%)	57.9	26.3	26.3	31.6	26.3	57.8
Specificity (%)	88.2	94.1	100.0	88.2	82.3	100
Positive predictive value (%)	84.6	83.3	100.0	75	62.5	100
Negative predictive value (%)	65.2	53.3	45.2	53.6	50.0	68.0
Positive likelihood ratio	4.9	4.5	+∞	2.7	1.5	+∞
Negative likelihood ratio	0.48	0.78	0.74	0.76	0.90	0.58
Kappa	0.45	0.20	0.25	0.19	0.08	0.57

Kappa = coefficient of concordance beyond chance.

## DISCUSSION

The results showed that it is possible to screen for stroke using a questionnaire about symptoms and previous medical diagnoses of stroke that can be applied by community health workers within the setting of primary care units and the Family Health Program. We used a screening instrument originally to evaluate the sensitivity of a simple screening question in comparison with a five-item questionnaire for diagnosing stroke in the MEMO Study.^22^ We found sensitivity of 57.8% for the diagnosis of stroke using only a single stroke question, compared with 65.8% in the original study. Limb weakness was the most frequent stroke symptom in both studies. However, the sensitivity was higher in our study (57.9% versus 39.5%). The least sensitive symptoms in the questionnaire were facial weakness, speech problems and impaired vision. These data are similar to the findings from the MEMO study, with the exception of impaired vision, which in their study was as sensitive as limb weakness. One possible explanation for this is that untreated ocular refractive errors are very common in Brazil due to the shortage of ophthalmologists in the most deprived areas and given that optometrists are prohibited from treating refractive errors.

Other studies worldwide have attempted to evaluate the prevalence of stroke in population-based samples using questionnaires. The Sicilian Epidemiological Study in 1987 was a two-phase epidemiological survey that evaluated the frequency and distribution of stroke and other neurological disorders. All the individuals who screened positive in phase one were evaluated by a neurologist in phase 2.^24,25^ In Latin America, a door-to-door survey was carried out in Junín, Argentina, in two stages: in stage 1, a responsible adult in the house, preferably the mother or spouse answered questions about stroke on behalf of all members of the family. Stage 2 included a complete neurological examination on all the individuals who screened positive in phase 1.^26^ Another door-to-door survey in Bolivia in 1994 studied prevalence of stroke in a two-phase survey. In phase 1, a sample from rural communities that was selected from 10 areas in Cordillera Province was screened door-to-door to identify individuals with possible stroke, using a questionnaire and simple tasks as the instruments. In phase 2, all the individuals who screened positive underwent a complete neurological examination.^27^ We also performed a two-phase study. However, in the second phase, a more detailed questionnaire was applied to all the adults aged over 35 years, and only the cases that participated in the validation study were examined by a neurologist. We also used the structure of the Family Health Program.

These two points made the present study less expensive than previous studies in which a neurologist examined all the individuals who screened positive. For a city like São Paulo, in which most of the neurologists are concentrated in the central and richer areas and are not available for research in primary care settings, this strategy seems more cost-effective. It can be used periodically to investigate changes in stroke frequency in neighborhoods of São Paulo, a city in which stroke is the third greatest cause of death, closely following coronary heart disease and cancer.^5,8^

One further important point is that our questionnaire is not very sensitive for diagnosing mild cases of stroke. One consequence of this is that the prevalence of stroke in this population was probably underestimated. However, this questionnaire is capable of selecting the most severe cases with greater degrees of disability, thus making it possible to identify the cases that need more rehabilitation support. Finally, we need to emphasize that our questionnaire was not applied as a "Questionnaire for Verifying Stroke-free Status",^28^ of the type that is used during the baseline phase of randomized controlled trials. Questionnaires like the stroke symptom questionnaire of the EMMA study and others used elsewhere^25,28^ are only to be applied for epidemiological surveys, epidemiological studies and clinical trials, by trained assistant researchers under neurological supervision. The use of this questionnaire for clinical purposes or by the hospital gatekeeper unit is strongly discouraged by the present authors.

## CONCLUSIONS

We conclude that it is possible to use a questionnaire applied by lay interviewers to identify cases of stroke using the structure of the Family Health Program. The data obtained could be used for strategic planning of primary care in the study areas.
